# Finding Freedom in Appalachia: Evaluating a Mental Health Intervention in West Virginia

**DOI:** 10.13023/jah.0701.02

**Published:** 2025-05-01

**Authors:** Elizabeth A. Claydon, Karen Haring, Jessica Haring, Kelsey Riggi, Beth Currence

**Affiliations:** West Virginia University; Libera, Inc.; Libera, Inc.; Libera, Inc.; Libera, Inc.

**Keywords:** Appalachia, intervention, lay counseling, mental health

## Abstract

**Introduction:**

Rural areas have limited capacity to provide comprehensive mental health treatment and resources. This creates the need for interventions to ensure individuals with mental health concerns have access to resources to improve their mental health.

**Purpose:**

The purpose of this mixed-methods evaluation was to assess the effectiveness and impact of Libera, a mental health program tailored for girls and women within the Appalachian region.

**Methods:**

Quantitative surveys, including validated measures for depression, anxiety, and eating disorder symptoms, and life satisfaction, were collected pre- and post-Libera intervention for participants. Simultaneously, annual surveys were sent to Listeners in addition to qualitative semi-structured interviews. Data were collected from August 2019 – November 2021. All data were analyzed from December 2021 – March 2022 using SAS JMP 16 for quantitative data and NVivo 14 for qualitative.

**Results:**

Participants showed significant improvements in depressive and anxiety symptoms, and life satisfaction following the Libera intervention. Disordered eating symptoms did not significantly improve. Similarly, Listeners showed changes from their training and experiences, with positive reactions, learning, behavior change, and effective results for them and their participants.

**Implications:**

This study provides evidence for Libera’s use among Appalachian women and girls. Participants experienced many improvements in mental health following completion of the Libera program. Additionally, Listeners leading groups experienced positive changes in learning and behavior. The results also indicate areas to continue strengthening the program with resources and training in specialized topics (such as eating disorders or Safe Zone training).

## INTRODUCTION

The Appalachian Region experiences significant mental health disparities, heightened by stigma, lack of access to care, social drivers of health, and cultural beliefs about mental health.^1^ Mental health stigma and barriers are pervasive, but there are some unique barriers which exist in Appalachia. Women in Appalachia have cited the cultural norms of self-reliance in rural areas, as well as a gender norm that women are not supposed to think negatively.^1^ These create additional internal barriers to care and can compound mental health concerns with guilt related to treatment seeking. Libera, a novel mental health intervention designed to connect girls and women to resources, has attempted to address these barriers and bridge the gap in West Virginia (WV).^2^

### Lay Counseling

Lay counseling has been defined as the “psychosocial support provided by staff or volunteers who do not have a mental health background or formal degree in counseling”.^3^ These lay counselors could be a variety of community members who are trained to support emotional health and provide interventions rather than diagnose or address complex comorbidities.

There have been several international examples of effective mental health interventions utilizing lay counselors. For example, interventions among adults with mood or anxiety issues in low- and middle-income countries,^4^ brief psychological interventions for mental disorders in Zimbabwe,^5^ and to address harmful drinking in India.^6^ In all cases, the interventions had high acceptability, often reducing the stigma of seeking treatment because individuals within a community felt comfortable receiving help provided by local peers rather than unfamiliar professionals.

Although these examples are global, lay counseling is highly translatable to the United States (U.S.) and is already used in some areas. Peer leaders and support services have been used as agents for addiction recovery, and a lay provider intervention has demonstrated effective delivery of cognitive behavioral therapy (CBT) to improve generalized anxiety disorder (GAD) among older adults.^7^, ^8^

Whether globally or nationally based, these types of programs improve the ability to reach underserved populations, bringing mental health support to people where they are.^9^ This approach also helps overcome cultural or trust-related barriers by training lay counselors within a population to deliver mental health interventions and provide resources – drawing from within a community encourages the acceptability of such interventions and promotes help-seeking behaviors. There is evidence that the use of lay counseling is not only acceptable and effective, but also cost-effective.^10^ Additionally, lay counselors could be the primary providers of interventions in low-resource areas, which can be translatable to rural and underserved populations within the U.S.^11^

### West Virginia Mental Health

Within WV, due to its rurality and battle with the opioid epidemic, children and adolescents are at an increasing need of mental health care and yet lack access to this care.^12^ In 2022, almost 2.8% of West Virginian children under the age of 18 were uninsured and almost 50% were enrolled in Medicaid.^13^ In WV, there are currently 6,126 children in state custody with the foster care population increasing by 57% in the last 10 years.^14^ This family dysfunction can create additional stress and mental health consequences. Teenage girls are also facing mental health challenges, with 63.6% of female-identifying teens feeling sad or hopeless.^15^ Similarly, women in WV rank consistently at or near the bottom in the U.S. for education, poverty, employment, domestic violence incidents/deaths, and incarceration.^16^ Depression lifetime prevalence in WV is the highest in the country, at 26.4% (the national prevalence of depression is 18.4%),^17^ and among women it is 29%.^18^ Anxiety prevalence is at 33%, higher than the US average of 28.2%.^19^

Additionally, other mental health issues such as eating disorders (EDs) will affect 9% of West Virginians across their lifetime, with women two times more likely to have an ED.^20^ The emotional and financial costs of these conditions are considerable, with caregivers providing 6 weeks of informal, unpaid care each year and an annual economic cost of $357.1 million to WV, primarily in the form of productivity losses.

These factors highlight the extensive need in WV for mental health interventions to fill the gaps and improve the health and well-being of girls and women, particularly.

## METHODS

### Libera Program Components

Libera uses the foundations of a lay counseling model and is a unique intervention that allows participants to share their stories in a safe environment. It is based on the idea that connection with oneself, with others, and with resources can help overcome barriers. LIBERA stands for *Listen, Illuminate, Believe, Envision, Reach, Alight.2* This acronym has a basis in the WV state motto: *Montani Semper Liberi*, or “Mountaineers are always free.” Libera uses the feminine form of *liberi*, meaning free, and their mission is to empower life transformation for West Virginian girls and women.

*Listen –* the opportunity to share stories uninterrupted and be listened to without judgment and with affirmation.

*Illuminate –* sharing stories helps girls and women illuminate the lies they believe about themselves and replace those lies with truth.

*Believe –* believing in one’s value and worth to empower participants to live the life they want and one that is truly free.

*Envision –* taking concrete steps to envision the life they want to live.

*Reach –* connecting participants to resources, groups, and nonprofits to help them reach their full potential.

*Alight –* sharing the experience with others and helping others to find their freedom.

The Libera program works over an eight-week period with weekly listening groups (eight total) alongside a workbook that educates and encourages participants to reflect and share their story in a judgment-free environment. Participants are recruited via schools, and potential Listeners are also required to participate in two eight-week listening groups as part of their training. Libera is a new model and approach to helping girls and women identify and talk about challenges that limit their freedom and applies a model for freeing them so that they can become successful in all aspects of their lives. These interventions occur in group settings at schools, libraries, and online (during the pandemic) across WV and specifically address providing available mental health resources in this rural state.

### Human Subjects

This study was approved by West Virginia University’s Institutional Review Board (IRB#: 1812375443). Informed consent was included in both quantitative and qualitative study processes. Regarding the inclusion of minors: parents had consented for their children and were aware of what the Libera program entailed. Therefore, a waiver of parental consent for the effectiveness evaluation was justified. Parents were notified of surveys and use of de-identified documents for analysis and had the ability to opt out for their child if they choose.

### Libera Participant Methods

Quantitative participant outcomes were completed and collected online through pre- and post-Libera intervention surveys at the beginning of groups and after the six-week groups completed. These were active from August 2019 – November 2021. Qualtrics software (Qualtrics, Provo, UT) was used to host and distribute the survey and no protected health information (PHI) was obtained. At the end of the survey, an option was provided for individuals to click a link to a separate survey to provide their email for a chance to win a $50 Amazon gift card. This ensured their email address was not connected with the data. See Appendix A for full survey. A total of 325 Libera participants completed the questionnaire prior to beginning listening sessions and 149 completed the post surveys.

### Libera Participant Measures

#### Anxiety (measured by GAD-7^21^)

Anxiety symptoms were measured with seven questions using the GAD-7, with a four-point Likert scale response from ‘Not difficult at all’ to ‘Extremely difficult.’ The GAD-7 is a screening tool centered on the individual’s experience over the past two weeks and includes questions like: “*Over the last two weeks, how much have you been bothered by any of the following problems?: Worrying too much about different things” or “Not being able to stop or control worrying*.” Scores are calculated with 0–3 points associated with each of the Likert scale responses, with 0 as least difficult and 3 as most. The score interpretation is: 0–4 indicate minimal anxiety, 5–9 mild anxiety, 10–14 moderate anxiety, and 15–21 severe anxiety. The GAD-7 has good psychometric properties, with excellent internal consistency, good test-retest reliability, as well as good validity. Our sample indicated good reliability (α =0.79).

#### Depression (measured by PHQ-9^22^)

The PhQ-9 consists of nine questions on a four-point Likert scale with answers related to frequency of feelings or behaviors (Not at all to Nearly Every Day). Similar to the GAD-7, the PHQ-9 is focused on mood and behavior over the last two weeks. Items include: “*Over the last two weeks, how much have you been bothered by any of the following problems?: “Feeling down, depressed or hopeless?”* or *“Feeling tired or having little energy?”* Points are assigned 0–3, with increasing points associated with increasing frequency. A total score of 1–4 indicates minimal depression, 5–9 indicates mild depression, 10–14 moderate depression, 15–19 moderately severe depression, and 20–27 severe depression. We found acceptable internal reliability for this scale in our study (α =0.77).

#### Life Satisfaction measured by Satisfaction with Life Scale (SWLS^23^)

This scale consists of five questions on a Likert scale response of 7 answers with answer choices ranging from ‘Strongly Disagree’ to ‘Strongly Agree’. Items include, for example: “*In most ways, my life is close to my ideal”* and *“If I could live my life over, I would change almost nothing.”* Points are allocated 1 to 7, with Strongly Agree having the most points. Scores from five to nine indicate that someone is extremely dissatisfied, 10–14 dissatisfied, 15–19 slightly dissatisfied, 20–24 slightly satisfied, 25–29 satisfied, and 30–35 extremely satisfied. It has high internal consistency and is a reliable scale for a range of different age groups. Within our sample, we found good internal reliability (α =0.80).

#### Disordered Eating (measured by SCOFF^24^)

This test has a total of five questions with dichotomous answers (yes/no). These relate to the acronym: *“Do you make yourself Sick because you feel uncomfortably full?”; “Do you worry you have lost Control over how much you eat?”; “Have you recently lost more than 14 pounds in a three-month period (original questionnaire asked for one stone, which was converted for American audiences)?”; “Do you believe yourself to be Fat when others say you are too thin?”;* and *“Would you say Food dominates your life?”* An answer of ‘yes’ to two or more questions indicates a potential eating disorder. Our sample showed acceptable internal reliability (α =0.75).

### Libera Listeners Measures & Methods

#### Survey

Libera Listeners received questionnaires during late summer of 2019, 2020, and 2021 hosted by Qualtrics software (Qualtrics, Provo, UT). The annual Listener survey consisted of four demographic questions, hours of Listener training they had completed, mental health first aid training status, how often they were provided with different resources or trainings, and specific Likert scale questions on the Listener training and accompanying handbook (answer choices ranged on a seven-point scale from strongly disagree to strongly agree). Example questions included: *“How frequently are you presented with additional trainings or resources apart from your initial training?”* and *“I feel that the Libera Listener Training prepared me for Libera group facilitation.”* See Appendix B for full questionnaire.

#### Interview

At the end of the Listener surveys, they were invited to participate in a more in-depth qualitative interview about their experiences using a guided qualitative interview protocol (See Appendix C). Ten interviews were conducted in 2019, nine in 2020, and five in 2021 (approximately a 29% interview response rate from the survey request). Those in 2019 were conducted on Libera buses on site; following the pandemic, all subsequent interviews took place via Zoom.

### Data Analysis Procedures

#### Quantitative

Descriptive analyses were run and expressed using frequencies and percentages for categorical variables and means and standard deviations for continuous variables. Pre- and post-surveys were matched using a participant ID (first two letters of middle name and two-digit day of birth. A matched-pairs t-test was conducted to compare the mean change on the GAD-7, PHQ-9, SWLS, and SCOFF from pre to post. All data analyses were conducted from December 2021 – January 2022 and were analyzed using SAS JMP 16.

#### Qualitative

E.C. conducted the interviews and took field notes throughout, along with trained graduate students. All participants consented for interviews to be recorded. In-person interviews were recorded via a digital voice recorder and manually transcribed. Zoom interviews were recorded via Zoom and auto transcribed. Thematic analysis was used to extract key recommendations and major themes. Across all years, students assisted with transcript corrections, codebook development, secondary coding, and compiling and presenting themes. Any divergences in coding were discussed and consensus was reached to ensure inter-coder reliability.

### Applying the Kirkpatrick model to Listener Training

The Kirkpatrick Model of Program Evaluation is a commonly used and valid structure to evaluate trainings.^25^ It includes four levels of evidence that are applied to trainings. These are: *Level 1 Reaction* – how participants respond to an intervention or training; *Level 2 Learning* – how participants gained skills or knowledge; *Level 3 Behavior* – how the participants changed their behavior based on the training provided; and *Level 4 Results* – was the training effective and had a return on investment. This model is used to structure the findings from the Listeners based on their Libera training.

## RESULTS

### Participants

Demographics for those matched from pre- to post-(N=85) indicated an average age of 34.89 (SD=18.12); the majority were white (N=75, 88.24%), and non-Hispanic (97.65%). Almost all identified as female (N =82, 96.47%) with the remainder identifying as nonbinary. See Table 1 for full demographics and Table 2 for demographics of the final sample. There was no difference between the demographics of the full sample versus final sample (age: p=0.6974; Hispanic: p=.4019; race: p=0.136).

#### Life Satisfaction

Libera participants had increased life satisfaction following the Libera intervention. The mean difference of the 85 participants who could be matched pre-to post-(M=0.98, SD=3.60, W=552) was significant (p=0.0072).

#### Depression

Scores from Libera participants showed a decrease in depression scores from pre- to post-intervention. The mean difference of the 85 participants matched pre to post showed a significant decrease in scores (M=−0.81, SD=3.33; W =−426 p=0.0137).

#### Anxiety

Based on scores from Libera participants, the average pre-intervention anxiety score decreased from pre- to post-intervention. The mean difference of the matched 85 participants was (M=−1.19, SD=3.22; W=−655, p=0.0013).

#### Disordered Eating

There were 106 participants (33.65%) who scored at or above 2 prior to the intervention (Libera), indicating those individuals had eating disorder symptoms. After the intervention, there were 39 participants (26.53%) who scored at 2 or above. When considering symptoms continuously of those matched pre- to post- (n=84), the mean difference (M=0.06, SD=0.87, W=26) was not significant (p=.5527).

### Listeners

All Listeners were female; the majority were white, and were in their late 40s, early 50s. Listeners participated in 11–19 hours of Listener training, and the majority had also completed a Mental Health First Aid training. See Table 3 for more detail on demographics and Table 4 for themes across all years.

#### Kirkpatrick Model

The findings from the Libera Listener evaluation are couched in the Kirkpatrick model levels below.^25^ This provides both a structure for the findings as well as strength of evidence based on the Libera training.[Fig f1-jah-7-1-24]

#### Level 1 Reaction

Level 1 of the Kirkpatrick model indicates how participants respond to an intervention or training.^25^ Listeners both qualitatively and quantitatively expressed positive responses to training, as well as challenges that could be addressed going forward. At the last time point, in 2021, most Listeners (N= 22; 62.86%) agreed or strongly agreed that the training prepared them for Libera group facilitation and 77.14% (N=27) agreed or strongly agreed that it prepared them to be an advocate for other women.

*Those [trainings] have been extremely helpful … Just having a comprehensive list of ways to approach situations – that I myself may not align with because it’s not my life experience.” (Libera Listener, 2020)*

Some suggestions included continuously revising and improving trainings and the handbook: “*By continuing to keep it updated according to needs we encounter while working with ‘clients.’” (Libera Listener, 2021)*

#### Level 2 Learning

The second level corresponded with gained skills and knowledge.^21^ Listeners who felt the mental health first aid prepared them well for a mental health crisis felt significantly better prepared for addressing depression in one-on-one Listening (p=0.0332).

Additionally, Listeners provided qualitative comments on aspects they learned that helped them be better Listeners such as:

*“Providing a doorway to allow participants to share without stigma.” (Libera Listener, 2021)*

They also discussed ways this training improved both their own lives and their work as a Listener:

*“I had always thought that you needed to keep yourself to complete exhaustion, emotionally and physically. But now I’ve learned that it’s okay to set boundaries and it isn’t less loving.” (Libera Listener, 2020)*

#### Level 3 Behavior

The third Kirkpatrick model addresses how the Listeners changed their behavior based on the training provided.^25^ One finding of note shows that Listeners who reported having higher levels of maintaining healthy boundaries (as indicated by the question: “During my service as a listener, I feel I can maintain healthy boundaries with those I listen to.”) were significantly more likely to have frequently referred to the Libera handbook (p=0.0127).

*“ This organization has given me a voice and confidence and a sense of worth and value even though I am a stay-at-home mom and often do not have many commonalities with working/professional women.” (Libera Listener, 2019)*

#### Level 4 Results

Level 4 considers if the training was effective and a return on investment*.25* Listeners across all years expressed the myriad benefits they felt in their own lives as well as in their ability to help others. They expressed that sharing stories in Libera is “*empowerment for the women.” (Libera Listener, 2019).* Others mentioned the impact in all areas of their life, including work:

“*Working in healthcare, I see my patients differently … it truly has impacted every interaction I have in my life. I wish I had seen it 20 years ago.” (Libera Listener, 2020)*

Most (N =30; 88.24%) Listeners also felt they could maintain their own self-care while being a Listener. Both qualitative and quantitative data have been used to refine the program, including the addition of a Safe Zone training for Listeners to better attend to LGBTQ+ participants, more eating disorder resources and material, and a revision of the teen workbook.

## IMPLICATIONS

These evaluation results illustrate that several key mental health indicators (such as depression and anxiety symptoms, and life satisfaction) significantly improved following the Libera intervention. These results are consistent with systematic reviews highlighting that lay counselors or paraprofessionals in the community can help improve mental health outcomes.^11^ Additionally, community interventions have been found to improve greater mental health literacy, which Libera fosters through awareness and conversation.^26^

Disordered eating was not significantly improved among participants, however more aspects about eating disorder awareness and resources were integrated following this evaluation period to address that limitation. However, this data was collected during the COVID-19 pandemic and there is longitudinal evidence to suggest there was an overall substantial increase in ED risk (measured by the SCOFF) during this time.^27^ There is also evidence to suggest that prevention and intervention efforts around disordered eating are successful in improving symptoms, especially when they promote emotional resilience.^28^ Since Libera already targets emotional resilience, pairing that with disordered eating resources should result in reduced symptoms over time. Future research will focus on follow-up in this area.

Listeners also responded positively to the training they received, felt that being a Listener gave them a purpose, and expressed how they felt additional benefits to being a Listener. Both quantitative and qualitative results indicated that not only did the Listeners find purpose helping others but also were able to foster better relationships personally and professionally due to the training and experience. This is consistent with research on how helping others, especially to find their own freedom, can establish a healthier sense of self and validate one’s identity and place in the world.^29^ These benefits were particularly important especially during the timing of this evaluation, which occurred over the COVID-19 pandemic, when individuals were more isolated from each other.

This study shows the possibilities for a unique lay counseling approach to connecting individuals with mental health resources within Appalachia. In 6-weeks, this intervention showed improvements in both participant and Listener lives; a short time span with volunteer Listeners can have an impact on the health and well-being of those in Appalachia.

### Strengths & Limitations

Due to inconsistencies with participants completing both the pre and post survey, only 85 individuals were able to be matched. Additionally, no further time points were selected to see progress on these measures. Gender identity was not collected in these surveys but will be collected in future studies. Attendance data was not collected, so we were not able to determine whether a certain ‘dose’ of the Libera intervention resulted in improvements. This is an area for future research. Convenience sampling was used for participant recruitment, which could introduce selection bias. Libera does not directly provide formal mental health care but focuses on listening to stories and connecting individuals with resources. However, Libera has a licensed counselor on staff should additional mental health assistance and connections be needed. By integrating both qualitative and quantitative methods, this study provides a more nuanced understanding of both participant and Listener experiences and outcomes. This will help to inform evidence-based practices to address mental health disparities in Appalachian communities.

### Future Directions

The Libera program has continued to expand throughout West Virginia and has some additional areas of focus, including incarcerated youth and youth aging out of the foster system. Some qualitative research has already been published regarding the suicidal ideations and experiences of incarcerated females, detailing themes that contributed to their risk for suicide.^30^ It also showed that almost ¾ of these incarcerated youth had seriously considered suicide.

Additional future directions include evaluating participant outcomes at multiple time points to understand the sustained changes longitudinally. The current research shows significant improvements in mental health in just a short period of time; future research will help to solidify those changes longitudinally.

**SUMMARY BOX**

**What is already known about this topic?**

Rural areas, particularly in Appalachia, face significant barriers to accessing mental health resources. There is a need for more interventions created by the community, for the community. Libera is one such example in the heart of Appalachia, using a model of Listeners to listen to and validate the stories of women and girls. This study showed improved participant outcomes in depression and anxiety scores as well as life satisfaction. Listeners also benefitted from the intervention, both in the ways they helped participants and in their own lives and relationships. This study indicates the possibility of using this intervention in more areas of Appalachia to address unmet mental health needs.

**What is added by this report?**

This evaluation provides program strengths, areas for improvement, and overall contributions to mental health and wellness in its target population. The primary hypothesized outcomes for participants were achieved, with increased life satisfaction and decreased depression and anxiety symptoms. Eating disorder symptoms did not significantly decrease. For Listeners (lay counselors), qualitative interviews gathered information on how Listeners became involved with Libera, their experience of it, and how it affected their own lives.

**What are the implications for future research?**

This study shows ways to connect individuals with mental health resources in Appalachia. There are significant benefits among participants and Listeners lives. Future research will continue to explore these benefits long-term and through expanded program aims.

## Supplementary Information



## Figures and Tables

**Figure 1 f1-jah-7-1-24:**
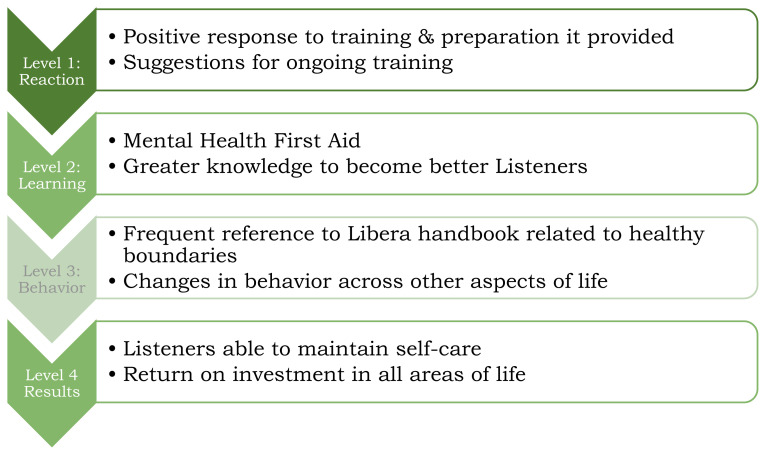
Diagram of Kirkpatrick Model with Libera Listener Quantitative & Qualitative

**Table 1:** Full Demographics

	Full Sample	
Characteristic	N (%)	M (SD)
**Life Satisfaction**		
Pre (n=325)		21.86 ± 7.37
Post (n=149)		22.68 (7.05)
**Depressive Symptoms**		
Pre (n=320)		8.07 ± 6.34
Post (n=149)		6.74 (5.78)
**Anxiety Symptoms**		
Pre (n=313)		7.52 ± 5.62
Post (n=148)		6.23 (5.45)
**Eating Disorders Symptoms**		
Pre (n=312)		1.13 (1.27)
Post (n=148)		1.01 (1.25)
**Age (n=318)**		35.83 (17.58)
**Race (n=322)**		
White	299 (92.86)	
Black	8 (2.48)	
Mixed	2 (0.62)	
Other	13 (4.04)	
**Hispanic (n=321)**		
Yes	14 (4.36%)	
No	321 (95.64%)	

**Table 2:** Libera Participant Demographics (Matched)

		Matched Sample	
Characteristic	N (%)	M ± SD	P-value
**Life Satisfaction**			0.0072*
Pre		22.06 ± 6.77	
Post		23.04 ± 6.74	
**Depressive Symptoms**			0.0297*
Pre		7.46 ± 6.00	
Post		6.64 ± 5.55	
**Anxiety Symptoms**			0.0013*
Pre		6.92 ± 5.25	
Post		5.73 ± 5.06	
**Eating Disorders Symptoms**			0.5527
Pre		0.98 ± 1.20	
Post		1.02 ± 1.26	
**Age**		34.89 ± 18.12	
**Race**			
White	75(88.24)		
Black	5 (5.88)		
Other	5 (5.88)		
**Hispanic**			
Yes	2(2.35%)		
No	83 (97.65%)		

**Table 3:** Listener Demographic Characteristics

	2019	2020	2021
	N = 10	N = 36	N = 38
Characteristic	N (%) or M(SD)	N (%) or M(SD)	N (%) or M(SD)
**Hours Libera Training**	15.3	19.1 (15.67)	17 (11.9)
**Age**	48.8	51.4 (14.25)	51.4 (13.4)
**Other Training Resources Provided** *			
Never	–	–	2 (5.4)
Occasionally	–	3 (8.33)	6 (16.22)
Sometimes	–	6 (16.67)	4 (10.81)
Frequently	–	24 (66.67)	22 (59.5)
Always	–	3 (8.33)	3 (8.11)
**Mental Health Training Complete**			
Yes	7 (70%)	28 (77.8)	24 (64.9)
No	3 (30%)	6 (16.67)	6 (16.2)
Unsure	–	2 (5.56)	7 (18.9)
**Race/ethnicity**			
White	10 (100%)	35 (97.22)	35 (94.6)
Other		1 (2.78)	2 (5.4)

NOTES:

* Question added in 2020

**Table 4:** Listener Themes Over Years of Evaluation

	Year				
	2019	2020	2021	Definition	Representative Quotes
**Training**					
Additional Needs Training	X	X	X	Any mention of additional training needed by Listeners to be better Listeners or lead groups more effectively.	“There were a couple non-binary [students] there – I don’t even know the correct term and don’t want to be politically incorrect - but almost gender-fluid? … That was tough because I didn’t want to insult them, I didn’t want to, you know, come across the wrong way, but for me, that’s a difficult concept to grasp.”
Feedback on Current Training	X	X	X	Any discussion of the training process for Listeners in Libera including resources provided.	“I can honestly say that the mental health first aid I thought was a very useful workshop to go through.”
**Benefits**					
Internal Benefits	X	X	X	Any discussion of benefits of listening that the Listeners have personally experienced in their own lives, including internal benefits, or being able to change their internal dialogues more positively.	“I don’t want to say that I wasn’t empathetic before, but definitely more empathetic, and less quick to judge I think too. Because as you listen to people, you find out that things are so much more complex, and less black and white, and that makes you hold judgement.”
External/Relational Benefits	X	X	X	Any discussion of benefits of Listeners that the Listeners have personally experienced including those related to having a shared experience with others and those related to jobs or relationships.	“As a people-pleaser, tell other people that I’m not in the mental state to take on your emotions, like help you with your emotions, and like put up that boundary without thinking that I’m like, mean or cold-hearted, but just knowing that it’s a healthy boundary that I can set.”
Involvement Process	X	X	X	Any discussion about how women got involved with Libera and how that involvement progressed and/or changed during their time with Libera, including their length of time involved with Libera and their experience leading groups.	“It just very much became a place that I felt included, and it was something that I absolutely knew that I needed to do.”
**Reasons for Joining**					
External Reasons for Joining	X	X	X	Any discussion about external reasons (such as friends involved with Libera) for joining Libera.	“It’s a great outreach, it doesn’t necessarily have the stigma of counseling, and it’s also free, which is incredible because so many women can’t afford counseling, it’s such a privilege.”
Internal Reasons for Joining	X	X	X	Any discussion about internal reasons (personal past experiences) for joining.	“First of all, it was the results. Like when you guys do the surveys and the end asking about hope, like I really did get a better sense of hope from it, and I really feel more empowered.”
**Challenges**					
Listening Challenges	X	X	X	Any discussion about general challenges that Listeners had experienced during their time as a Listener.	“Learning how to separate and knowing when to be able to distance yourself from someone who can become completely draining of not only your time, but of emotional resources.”
Training Challenges		X	X	Challenges related to training itself including mental health first aid or Libera training.	“Physically, just how long it was, and it also changes because I’ve said I’ve done two, and when no one wants to share or have something to say about the book, then it makes it kind of long and drawn out.”
Outreach	X			Any discussion about how Libera or Listeners help to support community mental health, recruit other Listeners, or participate in other community service.	“And now that I’m seeing them out in the community and listening to more of their stories and how their [sic] sharing their stories, it’s like, this has been a whole, just a way cool eye-opener, change of life, uh, empowerment for the women.”
Finding Purpose Through Libera		X	X	Any discussion related to how Listeners serve a purpose through Libera, including how they feel they can make a difference or are using Libera to make a difference or to help others.	“I’d spent a lot of my life thinking that I was alone, but I was just isolated. And I think it’s really, really important that women in Appalachia, specifically West Virginia, get this kind of help because we grow up in the same kind of cultural setting where we’re told to be certain things ... I just wanted to give back.” “I think that it filled a void that I was missing, which was connecting with other women, and connecting with parts of myself that I haven’t connected with.”
