# Supplementary material for: Finding Freedom in Appalachia: Evaluating a Mental Health Intervention in West Virginia
Source: J Appalach Health. 2025 May 1;7(1):22–46. doi: 10.13023/jah.0701.02 (PMC12143803; doi:10.13023/jah.0701.02)
Supplement: Supplementary file 1 [file Supplemental_Information(1).docx]

Appendix A: Pre & Post Participant Survey

Libera Pre-Assessment

Dear Libera Participant,

This letter is a request for you to take part in an evaluation of the Libera program. This project is being conducted by Dr. Elizabeth Claydon in the Department of Social & Behavioral Sciences at WVU School of Public Health. Your participation in this project is greatly appreciated and will take approximately 10 minutes to fill out the attached questionnaire. 

Your involvement in this project will be kept as confidential as legally possible. All data will be reported in the aggregate. You must be 16 years of age or older to participate. I will not ask any information that should lead back to your identity as a participant. Your participation is completely voluntary. You may skip any question that you do not wish to answer and you may discontinue at any time. This project has been acknowledged by West Virginia University’s Institutional Review Board.

I hope that you will participate in this research project, as it could be beneficial in understanding how Libera might help others like you. There will be a follow-up survey at the end of your participation with Libera that will also be voluntary for you to complete. At the end of the survey, there will be a list of mental health referrals and resources should you need them. 
Thank you very much for your time. Should you have any questions about this letter or this project, please feel free to contact Dr. Claydon at (304) 293-1900 or by e-mail at elizabeth.claydon@hsc.wvu.edu.
Thank you for your time and help with this project.

Sincerely,

Elizabeth Claydon, PhD, MPH, MS

*Assistant Professor*

Social & Behavioral Sciences

*West Virginia University* *School of Public Health*

Q2 I agree to participate in this project.

- Yes
- No

To help create an ID that will match to your post survey, we will ask you to answer the following two questions.

Please write your middle name.

________________________________________________________________

Please write the two-digit date you were born (example: May 4th would be 04).

________________________________________________________________

ID. Now, enter your ID by listing the first two letters of your middle name and the two-digit day of the month you were born on.  For example, if your middle name is Jennifer and you were born on the 3rd of the month, your ID would be: JE03. You will use this ID for this survey and your follow-up survey.

________________________________________________________________

Q3 What is your gender?

- Male
- Female
- Transgender (please specify: MTF, etc) ________________________________________________
- Nonbinary
- Prefer not to say

Q4 How old are you?

________________________________________________________________

Q5 Are you Hispanic or Latino/a?

- Yes
- No

Q6 What best describes your race?

- White
- Black/African American
- Asian
- American Indian, Alaskan Native
- Native Hawaiian, Pacific Islander, East Indian
- Other ________________________________________________

Q7.

What is your current height (feet)? .

What is your current height (inches)? .

Q8. What is your current weight (if you haven't weighed yourself recently, what is your best guess)?

________________________________________________________________

Q9 Below are five statements in which you may agree or disagree. Indicate your agreement with each item by selecting the appropriate bubble. Please be open and honest in your responding.

|  | Strongly Disagree | Disagree | Somewhat disagree | Neither agree nor disagree | Somewhat agree | Agree | Strongly agree |
| --- | --- | --- | --- | --- | --- | --- | --- |
| In most ways my life is close to my ideal. |  |  |  |  |  |  |  |
| The conditions of my life are excellent. |  |  |  |  |  |  |  |
| I am satisfied with my life. |  |  |  |  |  |  |  |
| So far I have gotten the important things I want in life. |  |  |  |  |  |  |  |
| If I could live my life over, I would change almost nothing. |  |  |  |  |  |  |  |

End of Block: Demographics

Start of Block: Mood

1. Over the last 2 weeks, how often have you been bothered by any of the following problems?

Little interest or pleasure in doing things?

- Not at all
- Several days
- More than half the days
- Nearly every day

2. Over the last 2 weeks, how often have you been bothered by any of the following problems?

Feeling down, depressed, or hopeless?

- Not at all
- Several days
- More than half the days
- Nearly every day

3. Over the last 2 weeks, how often have you been bothered by any of the following problems?

Trouble falling or staying asleep, or sleeping too much?

- Not at all
- Several days
- More than half the days
- Nearly every day

4. Over the last 2 weeks, how often have you been bothered by any of the following problems?

Feeling tired or having little energy?

- Not at all
- Several days
- More than half the days
- Nearly every day

5. Over the last 2 weeks, how often have you been bothered by any of the following problems?

Poor appetite or overeating?

- Not at all
- Several days
- More than half the days
- Nearly every day

6. Over the last 2 weeks, how often have you been bothered by any of the following problems?

Feeling bad about yourself - or that you are a failure or have let yourself or your family down?

- Not at all
- Several days
- More than half the days
- Nearly every day

7. Over the last 2 weeks, how often have you been bothered by any of the following problems?

Trouble concentrating on things, such as reading the newspaper or watching television?

- Not at all
- Several days
- More than half the days
- Nearly every day

8. Over the last 2 weeks, how often have you been bothered by any of the following problems?

Moving or speaking so slowly that other people could have noticed? Or so fidgety or restless that you have been moving a lot more than usual?

- Not at all
- Several days
- More than half the days
- Nearly every day

9. Over the last 2 weeks, how often have you been bothered by any of the following problems?

Thoughts that you would be better off dead, or thoughts of hurting yourself in some way?

- Not at all
- Several days
- More than half the days
- Nearly every day

End of Block: Mood

Start of Block: Anxiety

1. Over the last 2 weeks, how often have you been bothered by any of the following problems?

Feeling nervous, anxious, or on edge.

- Not at all
- Several days
- More than half the days
- Nearly every day

2. Over the last 2 weeks, how often have you been bothered by any of the following problems?

Not being able to stop or control worrying.

- Not at all
- Several days
- More than half the days
- Nearly every day

3. Over the last 2 weeks, how often have you been bothered by any of the following problems?

Worrying too much about different things.

- Not at all
- Several days
- More than half the days
- Nearly every day

4. Over the last 2 weeks, how often have you been bothered by any of the following problems?

Trouble relaxing.

- Not at all
- Several days
- More than half the days
- Nearly every day

5. Over the last 2 weeks, how often have you been bothered by any of the following problems?

Being so restless that it is hard to sit still.

- Not at all
- Several days
- More than half the days
- Nearly every day

6. Over the last 2 weeks, how often have you been bothered by any of the following problems?

Becoming easily annoyed or irritable.

- Not at all
- Several days
- More than half the days
- Nearly every day

7. Over the last 2 weeks, how often have you been bothered by any of the following problems?

Feeling afraid as if something awful might happen.

- Not at all
- Several days
- More than half the days
- Nearly every day

End of Block: Anxiety

Start of Block: Eating Behavior

1. Do you make yourself sick because you feel uncomfortably full?

- Yes
- No

2. Do you worry you have lost control over how much you eat?

- Yes
- No

3. Have you recently lost more than 14 pounds in a 3 month period?

- Yes
- No

4. Do you believe yourself to be fat when others say you are too thin?

- Yes
- No

5. Would you say that food dominates your life?

- Yes
- No

End of Block: Eating Behavior

Libera Post-Assessment

Dear Libera Participant,

The following survey is completely optional and inquires about your current mood, behaviors, and satisfaction with life. The purpose of this project is to understand how Libera is helping or can better help its participants. By understanding this, we can hope to improve or expand our programming. The information you provide is vital to helping others like you.

The evaluation of Libera is being overseen by myself, Dr. Elizabeth Claydon, of West Virginia University School of Public Health, in collaboration with Libera. The survey will take approximately 15 minutes to complete.

This survey is anonymous and cannot be linked back to you. You will have the option to provide your email address for entry to win a $50 Amazon gift card. Your email address will be kept confidential, and your data will never be linked to any identifying information such as that email address should you choose to provide it. Your participation in this survey is completely voluntary and you may choose to withdraw at any time. This project has been acknowledged by West Virginia University’s Institutional Review Board. Please contact elizabeth.claydon@hsc.wvu.edu with any questions.

If you agree to participate, please fill in the circle for ‘yes’ and continue to the survey.

Thank you so much for your time and willingness to help with this important project.

Sincerely,

Elizabeth Claydon, PhD, MPH, MS

I agree to participate in this project.

- Yes
- No

To help create an ID that will match to your pre survey, we will ask you to answer the following two questions.


Please write your middle name.

________________________________________________________________

Please write the two digit day of the month you were born (ex May 3rd would be 03).

________________________________________________________________

Now, enter your ID by listing the first two letters of your middle name and the two-digit day of the month you were born on.  For example, if you middle name is Jennifer and you were born on the 3rd of the month, your ID would be: JE03. You will use this ID for this survey and your follow-up survey.

________________________________________________________________

What is your gender?

- Male
- Female
- Transgender (please specify: MTF, etc) ________________________________________________
- Nonbinary
- Prefer not to say

How old are you?

________________________________________________________________

Are you Hispanic or Latino/a?

- Yes
- No

What best describes your race?

- White
- Black/African American
- Asian
- American Indian, Alaskan Native
- Native Hawaiian, Pacific Islander, East Indian
- Other ________________________________________________

What is your current weight (if you have not weighed yourself recently, what is your best guess)?

________________________________________________________________

Below are five statements in which you may agree or disagree. Indicate your agreement with each item by selecting the appropriate bubble. Please be open and honest in your responding.

|  | Strongly Disagree | Disagree | Somewhat disagree | Neither agree nor disagree | Somewhat agree | Agree | Strongly agree |
| --- | --- | --- | --- | --- | --- | --- | --- |
| In most ways my life is close to my ideal. |  |  |  |  |  |  |  |
| The conditions of my life are excellent. |  |  |  |  |  |  |  |
| I am satisfied with my life. |  |  |  |  |  |  |  |
| So far I have gotten the important things I want in life. |  |  |  |  |  |  |  |
| If I could live my life over, I would change almost nothing. |  |  |  |  |  |  |  |

End of Block: Demographics

Start of Block: Mood

Over the last 2 weeks, how often have you been bothered by any of the following problems?

Little interest or pleasure in doing things?

- Not at all
- Several days
- More than half the days
- Nearly every day

Over the last 2 weeks, how often have you been bothered by any of the following problems?

Feeling down, depressed, or hopeless?

- Not at all
- Several days
- More than half the days
- Nearly every day

Over the last 2 weeks, how often have you been bothered by any of the following problems?

Trouble falling or staying asleep, or sleeping too much?

- Not at all
- Several days
- More than half the days
- Nearly every day

Over the last 2 weeks, how often have you been bothered by any of the following problems?

Feeling tired or having little energy?

- Not at all
- Several days
- More than half the days
- Nearly every day

Over the last 2 weeks, how often have you been bothered by any of the following problems?

Poor appetite or overeating?

- Not at all
- Several days
- More than half the days
- Nearly every day

Over the last 2 weeks, how often have you been bothered by any of the following problems?

Feeling bad about yourself - or that you are a failure or have let yourself or your family down?

- Not at all
- Several days
- More than half the days
- Nearly every day

Over the last 2 weeks, how often have you been bothered by any of the following problems?

Trouble concentrating on things, such as reading the newspaper or watching television?

- Not at all
- Several days
- More than half the days
- Nearly every day

Over the last 2 weeks, how often have you been bothered by any of the following problems?

Moving or speaking so slowly that other people could have noticed? Or so fidgety or restless that you have been moving a lot more than usual?

- Not at all
- Several days
- More than half the days
- Nearly every day

Over the last 2 weeks, how often have you been bothered by any of the following problems?

Thoughts that you would be better off dead, or thoughts of hurting yourself in some way?

- Not at all
- Several days
- More than half the days
- Nearly every day

End of Block: Mood

Start of Block: Anxiety

Over the last 2 weeks, how often have you been bothered by any of the following problems?

Feeling nervous, anxious, or on edge.

- Not at all
- Several days
- More than half the days
- Nearly every day

Over the last 2 weeks, how often have you been bothered by any of the following problems?

Not being able to stop or control worrying.

- Not at all
- Several days
- More than half the days
- Nearly every day

Over the last 2 weeks, how often have you been bothered by any of the following problems?

Worrying too much about different things.

- Not at all
- Several days
- More than half the days
- Nearly every day

Over the last 2 weeks, how often have you been bothered by any of the following problems?

Trouble relaxing.

- Not at all
- Several days
- More than half the days
- Nearly every day

Over the last 2 weeks, how often have you been bothered by any of the following problems?

Being so restless that it is hard to sit still.

- Not at all
- Several days
- More than half the days
- Nearly every day

Over the last 2 weeks, how often have you been bothered by any of the following problems?

Becoming easily annoyed or irritable.

- Not at all
- Several days
- More than half the days
- Nearly every day

Over the last 2 weeks, how often have you been bothered by any of the following problems?

Feeling afraid as if something awful might happen.

- Not at all
- Several days
- More than half the days
- Nearly every day

End of Block: Anxiety

Start of Block: Eating Behavior

Do you make yourself sick because you feel uncomfortably full?

- Yes
- No

Do you worry you have lost control over how much you eat?

- Yes
- No

Have you recently lost more than 14 pounds in a 3 month period?

- Yes
- No

Do you believe yourself to be fat when others say you are too thin?

- Yes
- No

Would you say that food dominates your life?

- Yes
- No

End of Block: Eating Behavior

Appendix B: Listener Annual Survey

Dear Libera Listener, This letter is a request for you to take part in an evaluation of the Libera program. This evaluation is being conducted by Dr. Elizabeth Claydon in the Department of Social & Behavioral Sciences at WVU School of Public Health. Your participation in this project is greatly appreciated and will take approximately 10 minutes to fill out the attached questionnaire. This serves as a consent form for people ages 18 and older. Your parent or legal guardian has already signed a consent form. However, we also need you to agree to participate or not to participate in this survey. Please read this form carefully and indicate whether you agree or do not agree to participate. The fact that your parent previously signed a form does NOT obligate you to agree to participate. Your involvement in this project will be kept as confidential as legally possible. All data will be reported in the aggregate. You must be 18 years of age or older to participate. I will not ask any information that should lead back to your identity as a participant. Your participation is completely voluntary. You may skip any question that you do not wish to answer and you may discontinue at any time. West Virginia University's Institutional Review Board approval of this project is on file. There are no known or expected risks from participating in this evaluation study. There are no special fees for participating in this study. While you may not receive any direct benefit from this study, I hope that you will participate in this evaluation project, as it could give Libera suggestions about how they are able to improve the training of Libera Listeners. Should you have any questions about this letter or the evaluation project, please feel free to contact Dr. Claydon at (304) 293-1900 or by e-mail at elizabeth.claydon@hsc.wvu.edu. For information regarding your rights as a research subject, to discuss problems, concerns, or suggestions related to the research, to obtain information or offer input about the research, please contact the West Virginia University Office of Research Integrity and Compliance at (304) 293-7073. Thank you for your time and help with this project. Sincerely, Elizabeth Claydon, PhD, MPH, MS Assistant Professor Social & Behavioral Sciences West Virginia University

I agree to participate in this study.

- Yes (1)
- No (2)

I am at least 18 years old.

- Yes (1)
- No (2)

End of Block: Consent

Start of Block: Demographics

Enter an ID by listing the first two letters of your middle name and the two-digit day of the month you were born on.  For example, if your middle name is Jennifer and you were born on the 3rd of the month, your ID would be: JE03. You will use this ID for this survey and your follow-up survey.

________________________________________________________________

What is your sex?

- Male (1)
- Female (2)
- Transgender (please specify: MTF, etc) (3) __________________________________________________

How old are you?

________________________________________________________________

Are you Hispanic or Latino/a?

- Yes (1)
- No (2)

What best describes your race?

- White (1)
- Black/African American (2)
- Asian (3)
- American Indian, Alaskan Native (4)
- Native Hawaiian, Pacific Islander, East Indian (5)
- Other (6) __________________________________________________

End of Block: Demographics

Start of Block: Libera Listener Training

How many hours of Libera Listener training have you completed?

________________________________________________________________

How frequently are you presented with additional trainings or resources apart from your initial training?

- Never (1)
- Occasionally (2)
- Sometimes (3)
- Frequently (4)
- Always (5)

Have you completed the Mental Health First Aid training?

- Yes (1)
- No (2)
- Unsure (3)

Please indicate your agreement with the following statement: I feel that the Mental Health First Aid training prepared me to handle a mental health crisis.

- Strongly Disagree (1)
- Disagree (2)
- Somewhat disagree (3)
- Neither agree nor disagree (4)
- Somewhat agree (5)
- Agree (6)
- Strongly agree (7)

End of Block: Libera Listener Training

Start of Block: Libera Listener Training Assessment

Below are statements related to your initial Libera Listener Training in which you may agree or disagree. Indicate your agreement with each item by selecting the appropriate bubble. Please be open and honest in your responding.

The Libera Listener training handbook is...

|  | Strongly disagree (1) | Disagree (2) | Somewhat disagree (3) | Neither agree nor disagree (4) | Somewhat agree (5) | Agree (6) | Strongly agree (7) |
| --- | --- | --- | --- | --- | --- | --- | --- |
| organized. (1) |  |  |  |  |  |  |  |
| easy to understand. (2) |  |  |  |  |  |  |  |
| a resource I often refer to. (3) |  |  |  |  |  |  |  |

I feel that the Libera Listener training...

|  | Strongly disagree (1) | Disagree (2) | Somewhat disagree (3) | Neither agree nor disagree (4) | Somewhat agree (5) | Agree (6) | Strongly agree (7) |
| --- | --- | --- | --- | --- | --- | --- | --- |
| prepared me for Libera group facilitation. (1) |  |  |  |  |  |  |  |
| prepared me for Libera one-on-one listening. (2) |  |  |  |  |  |  |  |
| is useful in other settings apart from Libera groups or one-on-one listening. (3) |  |  |  |  |  |  |  |
| made me more knowledgeable about community resources. (4) |  |  |  |  |  |  |  |
| made me more knowledgeable about state-wide resources. (5) |  |  |  |  |  |  |  |
| prepared me to be an advocate for other women. (6) |  |  |  |  |  |  |  |
| prepared me for ethical decision making. (7) |  |  |  |  |  |  |  |

End of Block: Libera Listener Training Assessment

Start of Block: Participation

Below are statements related to your service as a Libera Listener in which you may agree or disagree. Indicate your agreement with each item by selecting the appropriate bubble. Please be open and honest in your responding.

While facilitating one-on-one or group meetings, I feel I am or would be able to help those I listen to with their needs related to:

|  | Strongly disagree (1) | Disagree (2) | Somewhat disagree (3) | Neither agree nor disagree (4) | Somewhat agree (5) | Agree (6) | Strongly agree (7) |
| --- | --- | --- | --- | --- | --- | --- | --- |
| Sexual Assault (1) |  |  |  |  |  |  |  |
| Intimate Partner Violence (2) |  |  |  |  |  |  |  |
| Finances (3) |  |  |  |  |  |  |  |
| Anxiety (4) |  |  |  |  |  |  |  |
| Depression (5) |  |  |  |  |  |  |  |
| Bullying (6) |  |  |  |  |  |  |  |
| Shame (7) |  |  |  |  |  |  |  |
| Lack of Direction (8) |  |  |  |  |  |  |  |
| Self-harm (9) |  |  |  |  |  |  |  |
| Religion (10) |  |  |  |  |  |  |  |

During my service as a listener...

|  | Strongly disagree (1) | Disagree (2) | Somewhat disagree (3) | Neither agree nor disagree (4) | Somewhat agree (5) | Agree (6) | Strongly agree (7) |
| --- | --- | --- | --- | --- | --- | --- | --- |
| I feel I can maintain healthy boundaries with those I listen to. (1) |  |  |  |  |  |  |  |
| I maintain my own self-care. (2) |  |  |  |  |  |  |  |

End of Block: Participation

Start of Block: Open-Ended Assessment

Without the use of names or identifiable information, can you briefly describe a time when you felt that you didn’t have the skills or knowledge necessary to help the woman you were listening to?

________________________________________________________________

________________________________________________________________

________________________________________________________________

________________________________________________________________

________________________________________________________________

Without the use of names or identifiable information, can you briefly describe a time when you felt you were most confident in your abilities as a listener?

________________________________________________________________

________________________________________________________________

________________________________________________________________

________________________________________________________________

________________________________________________________________

What are the most successful aspects of the Libera Listener training and training guide?

________________________________________________________________

________________________________________________________________

________________________________________________________________

________________________________________________________________

________________________________________________________________

How can the Libera Listener training or training guide be improved?

________________________________________________________________

________________________________________________________________

________________________________________________________________

________________________________________________________________

________________________________________________________________

What has been the most positive aspect of your service as a Listener?

________________________________________________________________

________________________________________________________________

________________________________________________________________

________________________________________________________________

________________________________________________________________

How can your experience as a listener be improved?

________________________________________________________________

________________________________________________________________

________________________________________________________________

________________________________________________________________

________________________________________________________________

Appendix C: Listener Interview Guide

Interview Guide:

Libera Listeners

**Description of the Project:**

The purpose of this study is to better understand the experiences of Libera Listeners. By understanding the unique aspects of the listening experience, we can learn how to better help the Libera organization, its Listeners, and the women they serve.

**Informed Consent:**

This interview will be kept completely confidential and all data will be de-identified. In order to ensure that any information you share today cannot be linked back to you, if you do mention any identifying information in this interview it will be removed from any transcripts before the data is examined. Thus, your information will always only be identified by a study ID. Your participation in this interview is completely voluntary and you may choose to withdraw at any time. This study has been filed with West Virginia University’s Institutional Review Board and approval has been obtained. If you have any questions as we go through the interview today, please don’t hesitate to ask. If there are any questions that I ask that you do not feel comfortable answering, you are under no obligation to answer them.

The interviews will take place in person in a private setting on the Libera buses. For purposes of this research, these interviews will be audio-recorded and then transcribed. Even after finishing the interview, if you do not feel comfortable with us keeping your data, you can ask that your audio-recording be deleted. No notes written by the interviewer during the interview will be kept either in that case.

***Interviewer prompt:*** Do you have any questions before we begin the interview? (If no, then: Okay, let’s get started then. I am going to start recording now.

1) Let’s get started with a general discussion about how you became a Libera Listener. What motivated you to join the organization?

*Probe: How did you learn of the organization’s existence?*

*Probe: How long have you been a Listener/how many groups have you led?*

2) What are some of the biggest challenge you have faced as a Libera Listener?

*Probe: How do you overcome such challenges?*

*Probe: How can Libera help you overcome such challenges?*

3) What benefits do you feel that you receive as a result of being a Libera Listener?

*Probe: What do you get out of the listening experience?*

4) How has becoming a Listener or being involved with Libera changed your life?

*Probe: What are some aspects of yourself or your life that have changed since becoming a Listener?*

5) What aspects of the Libera Listener training were the most helpful?

*Probe: What aspect of the training was the most challenging?*

6) Are you currently involved with other voluntary organizations?

*Probe: Have you been involved with other voluntary organizations in the past?*

*Probe: What types of voluntary organizations are you involved in (church-based, community-based, service through work or child’s school, etc.)*

7) Is there anything else related to this topic that you would like to share with me?

8) Do you have any questions for me?
